# Dance Performance in New Rural Areas Based on 3D Image Reconstruction Technology

**DOI:** 10.1155/2022/7122053

**Published:** 2022-05-27

**Authors:** Li Xie

**Affiliations:** College of Music and Dance, Yulin Normal University, Yulin 537000, Guangxi, China

## Abstract

Because of the special ecological environment and humanistic atmosphere in new rural areas, excellent regional dance art has been created. Through computer-aided technology, the essence of dance art in rural areas can be reconstructed and displayed. Therefore, based on 3D image reconstruction technology, this paper obtains the dance data of southeast Guangxi and puts forward the dance display scheme of new rural areas. Through acquisition of image information and image matching algorithm, the dance pose is estimated, and the extracted dance sequence is simplified by 3D reconstruction and mapped by texture. In addition, extraction effect of data set, comparison of dance similarity, and user authenticity score were used to test the five types of dance, which provides ideas for the inheritance and development of traditional folk dance culture.

## 1. Introduction

With the acceleration of the process of socialist modernization, the dance culture is gradually changing. If the development of traditional folk dance stagnates, it will inevitably be eliminated by society and the times, thus being marginalized and disappearing, especially for new rural areas. At the same time, the system of traditional dance culture has attribute of geographical structure, cultural, and social value which directly influence the development direction and mode of dance culture [1, 2].

At present, computer-aided technology already exists to help ethnic dance artists to carry out dance training and inheritance. Researchers express people's behavior, thoughts, and feelings through extracting, organizing, and artistic processing of human actions, but the movements of human limbs move in complex 3D space which is difficult to express it as simply and intuitively as the score of recorded music [3, 4]. Therefore, dance has always been regarded as an art that can only be understood but difficult to express. Computer-aided technology on dance refers to the form of capturing dance movements and abstracting them into models by means of 3D visual sensors and behavioral sensors. Through analysis, processing, and research, we finally create a dance form that is suitable for the preservation of movement information, so as to make scientific analysis and research on dance, sum up experience rules, and improve the skills of dance art [5, 6]. More importantly, it can record the characteristics of national dance art and contribute to the inheritance of cultural heritage.

As of late, with the turn of events and advocacy of profound learning, artificial brain networks have been effectively applied to the age of dance developments [7, 8]. The conspicuous benefit of embracing profound learning for dance age is that they can straightforwardly remove progressed highlights from crude information (sound, movement catch, and so forth). In addition, deep neural network can create new dance movements but with some problems in the dance generation algorithm based on deep learning. For example, because of the end-to-end model, the front and back frames of the generated dance may not be smooth, which will make the visualization effect of the generated dance worse. Dance data often come from the real world, so it is necessary to extract continuous data of dance pose by using human pose estimation technology [9, 10]. Therefore, applying 3D reconstruction technology to the dance field in new rural areas can promote the spread and protection of Chinese dance culture to a certain extent.

## 2. Scheme of Dance Display in New Rural Areas

### 2.1. 3D Image Reconstruction Technology

At present, computer graphics has entered the 3D era, and 3D graphics are everywhere. Virtual reality, scientific visualization, and 3D animation have become the three main research directions of computer graphics in recent years, and their technical core is 3D modeling and 3D reconstruction, which is the reverse process of camera imaging process. In other words, the central issue of 3D remaking innovation from a specific pixel point in the direction framework to a spatial point on the planet coordinate framework is the means to get the profundity data of the objective scene or article. Under the condition that the profundity data of the scene is known, the 3D remaking of the scene can be acknowledged distinctly through the enrollment and combination of point cloud information [11, 12].

#### 2.1.1. Introduction of Process

**Figure 1:**
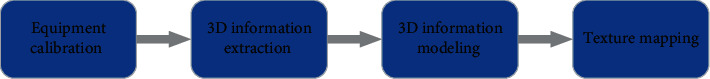
Process of image-based 3D reconstruction.

The acquisition of the depth information of the target object can be divided into passive measurement and active measurement [13, 14]. Active measurement refers to the use of 3D modeling software (such as Maya, 3D MAX, and CAD) and devices (such as scanners) to realize 3D reconstruction. The technology of reconstructing 3D models of real objects by this method is mature, but active operation is complicated. And there seems to be poor reconstruction effect and low efficiency of complex objects. Passive measurement is commonly referred to as image-based method, whose accuracy is relatively low, and the algorithm implementation is relatively complex, but it only needs less equipment and has less restrictions on the reconstructed objects. Image-based 3D reconstruction generally utilizes the impression of the general climate; for example, normal light utilizes the camera to get the picture and afterward works out the three-layered spatial data of the item through a particular calculation. It mainly consists of three phases as shown in Figure 1.

It includes three stages, that is, camera calibration, 3D information extraction, and texture mapping, where the most important part in the reconstruction is the 3D information extraction stage.

#### 2.1.2. 3D Coordinate Extraction

**Figure 2:**
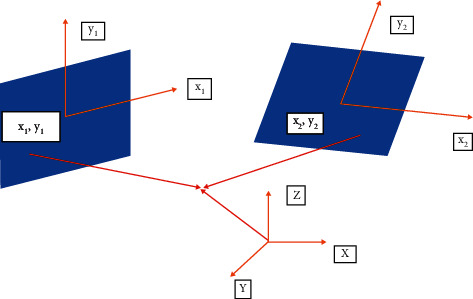
Principle of coordinate extraction.

For the computer, the change of angle is particularly complicated in the geometric calculation. The simplest geometric situation is that the camera image is located in the same plane, and those that are not in the same plane can be converted into the same image plane by linear transformation and reprojection, which is called image correction [15]. Stereo vision with multiple cameras under fixed illumination is also called motion recovery structure, and its basic principle is shown in Figure 2.

The purpose of 3D reconstruction is to uniquely determine the 3D coordinates (*X*, *Y*, *Z*) of point P by (*x*_1_, *y*_1_) and (*x*_2_, *y*_2_). So how to choose the most suitable matching point is tricky. Although the parallax results reflect the 3D positional relationship of the scene, there is still a slight gap between the parallax of some pixels and the standard value [16].

#### 2.1.3. Scheme of Dance Display in New Rural Areas

**Figure 3:**
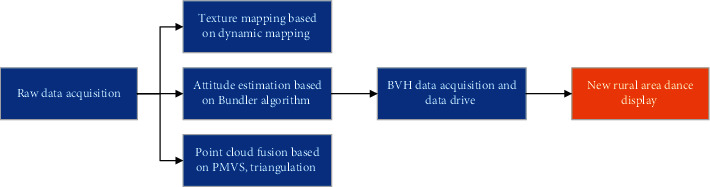
Scheme dance display in new rural areas.

Considering the complexity of the character model, the multiview stereo vision reconstruction algorithm based on feature points is used to reconstruct the character model. Firstly, the anthropomorphic model is used to experiment, and the rules of reconstructing the character model are explored. Then, the existing reconstruction algorithms are studied and improved, and a relatively perfect reconstruction algorithm is generated. According to experiences, the standard of obtaining data is determined through many comparative experiments [17]. After multiview stereo reconstruction, the anthropomorphic model is generated, the reconstruction effect of the anthropomorphic model is observed, and the model is modified appropriately to complete the establishment of the role model. In addition, texture mapping is carried out after the 3D character model is established to form a realistic model. The specific scheme is shown in Figure 3.

## 3. Implementation Process

### 3.1. Estimation Dance Pose

#### 3.1.1. Image Acquisition

In order to reconstruct the character model more easily, many factors should be paid attention in the process of photographing. In terms of light, try not to use flash and choose a room or passage with suitable light. If the light is insufficient, flash can be used to hit the ceiling or the back, so that the light can be evenly distributed. If the flash hits the object or the sunlight shines on one side of the object, the reflection will be formed on the surface of the object, which will easily lead to less matching points of the feature points of the photographed object, and the reconstruction cannot be completed.

**Figure 4:**
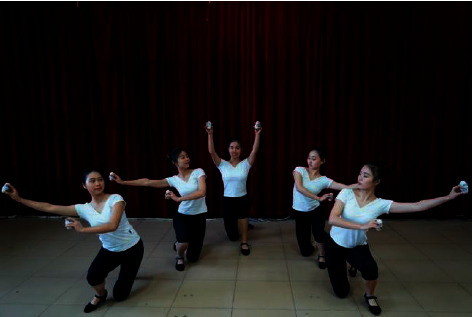
Images of folk dance in southeast Guangxi.

During shooting, it is necessary to avoid blurring the photos. The quality of the photos determines the number of point clouds in the reconstructed model and the workload to be modified after reconstruction. Moreover, it is forbidden to focus and change the exposure when taking the same group of photos, that is, choose the same camera to take pictures or take pictures by camera array.  Figure 4 shows the shooting of folk dances in southeast Guangxi. Each photo should be completely captured for objects. If there are incomplete characters or objects in the photos, it may lead to less matching of feature points, which may lead to the invalidation.

For the choice of the number of photos, we take the method of multiple photos to shoot each group of objects. The first angle was the flat angle, where one photo was taken every 18° on the flat angle and a total of 20 photos were collected. The second angle was looking down at 30°, where one photo was taken every 30°, and a total of 12 photos were collected. The third angle was looking down at 45°, where one photo is taken every 45°, and a total of 40 photos of 8 kinds are collected, which constitute a set of experimental image data. The angle moved by the photos can be roughly estimated without accurate measurement. In addition, when the photographed object is complex or the photographed image is reconstructed and some objects are missing, the number of photos can be added appropriately.

#### 3.1.2. Image Matching

In this paper, the SIFT-based image matching method is used, and the RANSAC algorithm is used to eliminate noise. In the field of target detection, the objects in the image are usually different in distance and size. The purpose of scale theory is to build a space where many details can be clearly seen at low scale and outlines can be seen at high scale. The scale space of the image is defined as follows:

(1) $$\begin{array}{c} L\left(x,y,\sigma \right)=G\left(x,y,\sigma \right)*I\left(x,y\right). \end{array}$$

Among them, *G*(*x*, *y*, *σ*) is a Gaussian function with variable scale.

(2) $$\begin{array}{c} G\left(x,y,\sigma \right)=\left(\frac{1}{2\pi \sigma^{2}}\right)e^{-\left(x^{2}+y^{2}\right)/2\sigma^{2}}, \end{array}$$

where (*x*, *y*) represents the spatial coordinates and *σ* represents a scale coordinate. The blur degree of the image is determined by *σ*, high value of *σ* corresponds to the general feature of the image, that is, the low resolution of the image; and low value of *σ* corresponds to the detail feature of the image, that is, the high resolution of the image.

SIFT takes image pyramid to build scale space, and DOG is a special image pyramid as follows:

(3) $$\begin{array}{c} D\left(x,y,\sigma \right)={\left(G\left(x,y,k\sigma \right)-G\left(x,y,\sigma \right)\right)}^{*}I\left(x,y\right)=L\left(x,y,k\sigma \right)-L\left(x,y,\sigma \right). \end{array}$$

In order to find out the extreme point of scale space, it is necessary to compare the sample point with all points of the same scale (i.e., image space) and adjacent scale (i.e., scale space). If the sample point is the maximum or minimum of all points, then the sample point is the extreme point of scale space. By marking points with 8 points of the same scale and 18 points of adjacent scale, a total of 26 points are compared. If the marked point is the maximum or minimum value, it can be guaranteed that it is the extreme point in both image space and scale space, and then the marked point is one of the feature points in this scale. In the process of extreme value comparison, it is impossible for the first and last two layers of each group of images to compare extreme values.

Using DOG pyramid will produce strong edge correspondence, and some points obtained by extreme value comparison may be edge response points. Therefore, in order to select feature points more accurately, it is necessary to use the derivative of spatial scale function and Hessian matrix to achieve subpixel accuracy, which can further eliminate feature points and edge response points that do not meet the contrast requirements.

The spatial scale function is shown in the following formula:

(4) $$\begin{array}{c} D\left(x,y,\sigma \right)=D\left(x,y,\sigma \right)+\left(\frac{\partial D^{T}}{\partial x}\right)x+\frac{1}{2x^{T}}\left(\frac{\partial^{2}D}{\partial x^{2}}\right)x. \end{array}$$

Deriving it and making it 0, the accurate position $\hat{x}$ can be obtained.

(5) $$\begin{array}{c} \hat{x}=-\left(\frac{\partial^{2}D^{-1}}{\partial x^{2}}\right)\left(\frac{\partial D}{\partial x}\right). \end{array}$$

In the feature points that have been compared with extreme values in scale space before, the feature points and edge response points that do not meet the contrast requirements are eliminated.

Introduce $\hat{x}$ into *D*(*x*, *y*, *σ*) and take the first two terms of the space scale function:

(6) $$\begin{array}{c} D\left(\hat{x}\right)=D\left(x,y,\sigma \right)+\frac{1}{2}\left(\frac{\partial D^{T}}{\partial x}\right)\hat{x}. \end{array}$$

If $\left|D\left(\hat{x}\right)\right|\langle 0.03$, the feature point is discarded.

The edge response points are eliminated by the principal curvature. If the feature points compared by extreme values have a larger principal curvature in the direction parallel to the edge and a smaller principal curvature in the direction perpendicular to the edge, the feature points should be discarded. A 2 × 2 Hessian matrix of *H* is defined as $H=\left[\begin{array}{cc} D_{xx} & D_{xy}\\ D_{xy} & D_{yy} \end{array}\right]$.

The principal curvature of the spatial scale function is proportional to the eigenvalue of the Hessian matrix, so that *α* is the maximum characteristic value, *β* is the smallest eigenvalue, and then

(7) $$\begin{array}{c} Tr\left(H\right)=D_{xx}+D_{yy}=\alpha +\beta ,\\ \text{Det}\left(H\right)=D_{xx}D_{yy}-{\left(D_{xy}\right)}^{2}=\alpha \beta . \end{array}$$

Make *σ*=*γβ*^2^, then

(8) $$\begin{array}{c} \text{Det}\left(H\right)=\frac{\left(\alpha +\beta^{2}\right)}{\alpha \beta }=\frac{{\left(\gamma \beta +\beta \right)}^{2}}{\gamma \beta^{2}}=\frac{{\left(\gamma +1\right)}^{2}}{\gamma }. \end{array}$$

If the ratio is greater than (*γ*+1)^2^/*γ*, remove it and make *γ*=10 in SIFT. The eliminated points are the detected SIFT feature points, and their positions have been determined. The direction parameters of each SIFT feature point are determined by the gradient direction.

(9) $$\begin{array}{c} m\left(x,y=\sqrt{{\left(L\left(x+1,y\right)-L\left(x-1,y\right)\right)}^{2}+{\left(L\left(x,y+1\right)-L\left(x,y-1\right)\right)}^{2}}\right). \end{array}$$

(10) $$\begin{array}{c} m\left(x,y=\sqrt{{\left(L\left(x+1,y\right)-L\left(x-1,y\right)\right)}^{2}+{\left(L\left(x,y+1\right)-L\left(x,y-1\right)\right)}^{2}}\right). \end{array}$$

Use (9) and (10) to find the modulus and direction of gradient in feature points (*x*, *y*), respectively.

Through the above calculation, all SIFT feature points in the image have been detected, and the feature area of each feature point can be determined by the location, scale, and direction of each feature point.

#### 3.1.3. Attitude Estimation

In computer vision, motion and structure reconstruction refer to recovering corresponding 3D information from 2D images or videos, including camera motion parameters and scene structure information. In this paper, we use Bundler based on the Levenberg–Marquardt algorithm. After detection and match of features, Bundle Adjustment can be based on the projection of all points in the image as the standard and the relative motion parameters of 3D points and the parameters of the camera are obtained at the same time [18]. It can match the observed image position with the predicted image position by minimum error that is expressed by the sum of squares of nonlinear functions:

(11) $$\begin{array}{c} x^{*}=\text{arg}\underset{x}{\text{min}}\sum_{i=1}^{k}f_{i}{\left(x\right)}^{2}. \end{array}$$

Minimizing error is realized by the nonlinear least square method, and formula (12) can be used to express the working process of Bundle Adjustment:

(12) $$\begin{array}{c} {\text{min}}_{a_{j},b_{i}}\sum_{i=1}^{n}\sum_{j=1}^{m}v_{ij}d{\left(Q\left(a_{j},b_{i}\right),x_{ij}\right)}^{2}, \end{array}$$

assuming that there are *n* 3D points in *m* shooting scenes. *V*_*ij*_ represents the mapping relation of point *i* on image *j*. If there exists mapping relation, *V*_*ij*_ is taken as 1; otherwise, it is 0. *a*_*j*_ is the vector parameterization of image *j*, and *b*_*i*_ is the parameterization of 3D points. *Q* is the predicted image bit of point *i* on image *j*, and *x*_*ij*_ is the observed image bit. The process of minimization is to find the minimum error match between predicted image bits and observed image bits.

### 3.2. Simplification of 3D Model

Mesh simplification is to reduce the precision of the model surface, reduce the number of triangular surfaces in the model as much as possible, and keep the shape of the original model as much as possible in the simplified model, which finally generate an approximate model of the original model. The approximate model basically keeps the features of the original model, including geometric features and visual features. However, the number of vertices and triangles is less than that of the original mesh. There are usually two kinds of constraints when generating the approximate model, namely, the triangle number constraint and the simplified error constraint [19, 20]. Through mesh simplification, a number of simplified models with different resolutions can be generated, and the real-time rendering can be accelerated. The technology of model simplification can make the storage, transmission, calculation, and display rendering of the model more effective, which can reduce the consumption of hard disk and memory and make the computer more effective, so as to achieve faster speed of rendering and execution.

In this paper, we use the subdivision level function provided by Zbrush to change the number of triangular faces, and the subdivision level function adopts the method of triangle folding. When the model is rendered, advanced subdivision is adopted, so that the rendering effect is better and more realistic. When the model is output, it is simplified and low-level subdivision is adopted, which can speed up the operation efficiency and facilitate the subsequent modification.

### 3.3. Texture Mapping

Texture mapping can be realized by scanning or digital photography, such as mapping with image processing software Photoshop or directly drawing texture on 3D surface in 3D drawing tools. This process specifically assigns a texture coordinate to each vertex on the polygon mesh, which is known as UV coordinates in the case of 2D; this process can be completed by explicitly assigning vertex attributes and manually editing in 3D modeling package, where the conversion from 3D space to texture space can also be realized by plane projection cylindrical mapping or spherical mapping application.

The dynamic mapping technology in Zbrush is also selected, which benefits from Zbrush's powerful drawing function. We can bake the texture in the photo directly to the 3D character model, draw the texture color on the character model through photos from different angles, then spread it through UV (equivalent to cutting the surface of the 3D model), and finally integrate the finished texture into a 2D texture map.

## 4. Test Results and Analysis

All our model experiments were trained on a NVIDIA 1080tigpu. In the generator training stage, we set the number of training rounds of the model to 10,000 rounds. The input dimension of the model is 35, the number of convolution layers of the encoder is 3, the maximum length of each convolution kernel is 5, the dimension of the decoder RNN is 1024, the dimension of Prenet is 256, the learning rate is set to 0.001, the gradient clipping threshold is set to 1, and the weight attenuation is set to 1e^−6^. Batchsize is set to 40, and seqlen is set to 125. In addition, the learning rate of the discriminator is 0.001, the weight attenuation is 1e^−6^. The training set takes 80% of dance data, and the rest are adopted by the test set, and the dance data were taken from Tea things, Qianbian Dance, Cup Dance, and other forms in southeast Guangxi.

### 4.1. Results Feature Extraction

In the stage of dance feature extraction, the influence of different data processing methods on the final loss function is analyzed. Without filtering out the wrong data, the loss value using the model characteristics will be greater than the loss value without using the model characteristics. As the dimension of dance data increases, the noise may also increase, but the motion features may be useful for the final generation effect of dance, so it is better to use interpolation function to supplement the missing values of dance data than not. It can be seen from Table 1 that whether to filter out the wrong data has the most important influence on the final result. As long as the key points of the wrong human posture are removed, the final result will be significantly enhanced.

### 4.2. Results of Dance Sequence Generation

The training set of the dance data set is preprocessed, and the dance sequence is segmented according to the settings of seqlen = 125 and batchsize = 40. The similarity between the corresponding dance sequence and the generated dance sequence is calculated. If a piece of dance has appeared in the previous training data, then directly calculate the similarity between the dance sequence and the real dance sequence to measure the actual generation effect of dance. The similarity of five dances in southeast Guangxi is compared, and the results are shown in Table 2.

As can be seen from Table 2, the similarity of dance display in southeast Guangxi after image-based 3D reconstruction is high, among which the Money whip dance is the highest, reaching 93.29%. However, the similarity of reconstructed Chikuma dance is relatively low, which may be due to the fact that it pays more attention to the simulation of animal behavior, and its recognition degree in dancers' modeling and movement arrangement is low.

### 4.3. Authenticity Score Results

The degree of reduction of the reconstructed dance was investigated. We invited 20 local observers to conduct scoring experiments and showed each observer 15 pieces of five different kinds of reconstructed dances. Each observer scores according to the fidelity of the dance where the highest score is 10 points and the lowest score is 0 points. The scores of each model are averaged according to the raters' scores of 15 videos, and finally the scores of all raters are averaged, so that the trueness scores of each model can be obtained.

**Figure 5:**
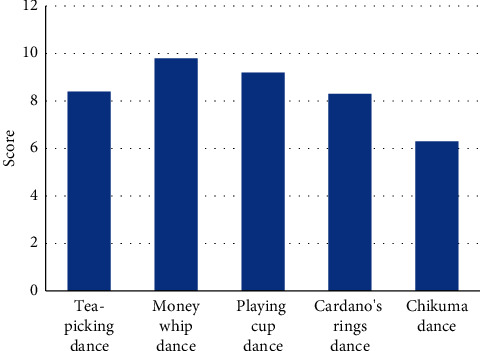
User's score of dance authenticity.

As can be seen from Figure 5, the score of users on the authenticity of dance in southeast Guangxi is quite consistent with the result of the similarity of dance sequences, with Tea-picking dance, Money whip dance, Playing cup dance, Cardano ring dance, Chikuma dance scoring 8.4, 9.8, 9.2, 8.3, and 6.3, respectively. Therefore, the dance display in new rural areas based on 3D image reconstruction technology has gained excellent user evaluation.

## 5. Conclusion

In this paper, aiming at the dance display in new rural areas based on 3D reconstruction, the 3 character reconstruction and texture mapping based on images are deeply studied, the process of dance display in southeast Guangxi under 3D reconstruction technology is introduced, and five types of local dance are selected for test and analysis. The results show that the pose sequence generated by the restructured dance model is reasonable, where there are fewer incorrect and unreasonable pose frames, and the similarity with the dance in the original area is high. In terms of users' evaluation, the dance generated by 3D reconstruction technology has a good effect in restoring the authenticity. Among the five dance types displayed, the reorganization effect of Qianbian Dance is the best, with a similarity of 93.29% and a user authenticity score of 9.8.

## Figures and Tables

**Table 1:** Extraction effect of dance data set.

Method	Train	Valid	Test
Frames not dropped	20.39	22.28	32.89
Interpolation	17.23	19.32	25.83
Frames dropped	11.09	12.20	16.36
Interpolation	9.89	11.90	14.23

**Table 2:** Comparison of dance similarity in southeast Guangxi.

Name of the dance	Similarity (%)
Tea-picking dance	87.49
Money whip dance	93.29
Playing cup dance	92.20
Cardano ring dance	89．20
Chikuma dance	68.76

## Data Availability

The dataset can be accessed upon request.
